# Sequence and origin of the Streptomyces intergenetic-conjugation helper plasmid pUZ8002

**DOI:** 10.1099/acmi.0.000808.v3

**Published:** 2024-06-05

**Authors:** Daniel E. Larcombe, Robyn E. Braes, James T. Croxford, James W. Wilson, David H. Figurski, Paul A. Hoskisson

**Affiliations:** 1 Strathclyde Institute of Pharmacy and Biomedical Sciences, University of Strathclyde, 161 Cathedral Street, Glasgow, G4 0RE, UK; 2 Villanova University, 800 Lancaster Avenue, Villanova, PA 19026, USA; 3 Department of Microbiology and Immunology, Columbia University, 701 W. 168 St., HHSC 1208, New York, NY 10032, USA

**Keywords:** conjugation, *Escherichia coli*, IncP plasmids, intergeneric, RK2, *Streptomyces*

## Abstract

Conjugation of plasmids from *Escherichia coli* is essential for the genetic manipulation of *Streptomyces* spp. To facilitate intergeneric conjugation from *E. coli* to *Streptomyces* the conjugative machinery required for genetic transfer is usually provided by the non-transferable helper plasmid, pUZ8002. Here we present the complete nucleotide sequence of pUZ8002, describe the previously undocumented creation process, and provide details of the sequence relative to the parental pUZ8 plasmid and another previously published pUZ8002 sequence.

## Data Summary

Raw sequencing reads are available on the lab Figshare: https://doi.org/10.6084/m9.figshare.252132681[1].https://doi.org/10.6084/m9.figshare.25213268.

## Introduction

*Streptomyces* are Gram-positive bacteria representing the largest genus in the phylum Actinomycetota. They are generally found as environmental isolates living in the soil and demonstrate a hyphal-like multicellular morphology [2]. *Streptomyces* are prolific producers of active secondary metabolites and many of these compounds are used clinically as antimicrobials and antitumour and immunoregulatory agents. Indeed, around two-thirds of antibiotic types currently used are produced by actinomycetes, predominantly *Streptomyces* [3]. Recent genome mining has revealed an even more expansive suite of silent biosynthetic gene clusters (BGCs) present in the genomes of *Streptomyces* species. These silent BGCs may be the key to finding new antimicrobials to overcome the rise of antimicrobial resistance [4,7]. Genetic manipulation can help both express these silent BGCs and improve the robustness or yield of production strains of *Streptomyces* currently used in industrial fermentations [8,10].

Unlike some other bacteria, *Streptomyces* are challenging to transform directly. Generally, the cell wall of *Streptomyces* must be removed first (forming protoplasts) to allow transformation at reasonable efficiencies [11]. Protoplast transformation can require optimisation for different *Streptomyces* species and imposes a great stress on the cells [12,14] and can potentially introduce mutations. To overcome this and expedite the process of genetic manipulation of *Streptomyces*, researchers generally rely on conjugation of genetic material from *Escherichia coli*. This conjugation requires a plasmid vector containing an *oriT* such as pSET152 or pMS82 [15,16], a methylation-deficient donor *E. coli* strain such as ET12567 [17] and a helper plasmid to provide the machinery required for conjugation. In *Streptomyces* molecular genetics, the helper plasmid is generally pUZ8002, which is widely reported to be derived from the well-characterised broad-host-range RK2 plasmid [18]. RK2 is a IncP-type extra-chromosomal plasmid first identified during an outbreak of *Pseudomonas aeruginosa* and *Klebsiella aerogenes* from the Burns unit of the Birmingham Accident Hospital, UK, in 1969 [19]. RK2 carries genes for tetracycline and kanamycin resistance, genes for self-replication such as *trfA*, and genes necessary for conjugation, including the *tra* and *trb* locus. RK2 also has its own *oriT*, which allows self-conjugation and origin of replication for autonomous replication [20,22]. There are many plasmids in the IncP group and pUZ8 is another example, originally isolated from a Spanish strain of *P. aeruginosa* [23]. Unlike RK2, pUZ8 has not been fully sequenced, but is known to be similar to RK2, with both plasmids carrying tetracycline and kanamycin resistance genes [24].

Limited details are available on the construction of pUZ8002, yet it is amongst the most widely used plasmid in *Streptomyces* molecular genetics. The first reference to pUZ8002 by name cites the plasmid source as ‘personal correspondence by Wilson, J., and Figurski, D. H.’ [18]. This paper indicates that pUZ8002 is an ‘RK2 derivative with defective *oriT*’ but no further details of the plasmid are given. This work by Paget *et al*. [18] appears to be the first published use of pUZ8002 as a helper plasmid to supply the conjugative machinery for movement of plasmids into *Streptomyces*. Currently one other sequence of pUZ8002 is available online under the GenBank accession number MN602278.1. This work aims to verify the sequence of pUZ8002 from our commonly used laboratory strains of *E. coli,* detail the process for the construction of pUZ8002 and determine the differences from RK2.

## Methods

### Plasmid extraction, transformation and sequencing

Plasmid DNA was extracted from *E. coli* using a PureYield plasmid miniprep system from Promega Uk Ltd (Hampshire, UK) following the manufacturer’s instructions. Competent DH5α cells for transformations were provided by New England Biolabs (Hertfordshire, UK) and transformations were carried out according to the manufacturer’s instructions. Plasmid sequences were obtained from Plasmidsaurus (Oregon, USA) using their Oxford Nanopore sequencing service.

### Bioinformatic analysis

Prokka [25] on the Galaxy web client was used to analyse and annotate the features of the plasmid sequence, selecting all default settings for bacteria (Galaxy version 1.14.6 [26]). Additional annotations were added manually from the previously published RK2 sequence [21,27]. All ORFs were checked manually via blastp and any alternative gene names are noted in the snapgene file (https://doi.org/10.6084/m9.figshare.25213268). Annotated plasmid sequence can also be found under GenBank accession number PP430156.

## Results and discussion

### Construction of pUZ8002

Although commonly attributed to being RK2 derived, pUZ8002 was actually constructed from the pUZ8 backbone. The key difference between pUZ8 and pUZ8002 is the inactivation of the *oriT* preventing self-conjugation. Four nucleotide changes are introduced at positions 46 858, 46 861, 46 862 and 46 863 (further details in Table 1) in the *oriT*, which eliminates the TraI-binding and *nic* site [28]. Destruction of the *nic* site prevents the covalent linkage of the 5′ end of the plasmid to TraI, preventing self-conjugation of the plasmid [21,28]. The method used to achieve this is the same as that detailed for the construction of pRK21761 from RK2 in [29]. In brief: the four point mutations were introduced directly into the pUZ8 parental plasmid via homologous recombination of two vectors either side of the *nic* site, which replaced the *nic* site with an XbaI site. These two vectors were then removed by digestion of pUZ8 with XbaI and religation, leaving a single XbaI site in place of the *nic* site. With pRK21761 this results in a 1000-fold decrease of self-conjugation efficiency while still allowing conjugation of other vectors containing functional *oriT* sequences [29,30].

**Table 1.** Comparison of pUZ8002 and the previously published RK2 sequence (GenBank BN000925.1). The four mutations introduced into pUZ8 to prevent self-conjugation are in bold [21,27]

Type	Length (bp)	Bases	ORF	Effect	Position in pUZ8002
Polymorphism	1	G to T	*klaB*	Ala162Glu	1 018
Polymorphism	1	G to T			2 235
Tn insertion	4 207		*merRTPCAD* TN3 disrupting RK2’s *kleD*		3 307–7 514
Tn deletion	4 949		RK2’s Tn1, which disrupted *klcB*, has been deleted, removing *tnpA* and *tnpR*		9 701
Insertion	1	A	*oriV*		11 493
Insertion	24		*oriV*	Additional TrfA binding site sequence detected in repetitive region	11 660–11 682
Polymorphism	1	T to A	*oriV*		11 964
Deletion	1	G	*tetR*	Frameshift at 175/184 in TetR	12 724
Polymorphism	1	T to G	*tetA*	Ile30Arg	13 338
Polymorphism	1	G to A	*tetA*	Val80Met	13 487
Polymorphism	2	AA to GG	*tetA*	Ile100Val	13 546–13 457
Polymorphism	2	CA to TG	*tetA*	Thr108Ala	13 573–13 574
Polymorphism	1	A to G	*tetA*		13 600
Deletion	785				1 4525
Insertion	75		GNAT domain containing N-acetyltransferase		14 526–14 601
Insertion	1	T			14 976
Deletion	1	T	*ssb*		16 284
Polymorphism	1	A to C			16 659
Polymorphism	1	C to G	*traX* (= *trbP* in RK2)	Pro35Ala	29 593
Polymorphism	3	CGC to GCG	Hypothetical protein	Pro88Arg Pro89Ala	3 0504–30 506
Tn deletion	2939		IS21 transposon that was in *aphA* gene is deleted		34 343
Deletion	1	G	Kanamycin resistance *aphA*	Frameshift from 146 to 161	34 761
Deletion	1	G	Kanamycin resistance *aphA*	Frameshift from 150 to 161	34 771
Insertion	2	CG	Kanamycin resistance *aphA*	Corrects frameshift from previous two deletions from 161	34 804–34 805
**Polymorphism**	**4**	**CctGCC to TctAGA**	***oriT* - TraI *nic* site**	**Introduces an XbaI site and inactivates the TraI cleavage (*nic)* site**	**46 858–46 863**
Insertion	1	T			48 696
Deletion	1	C			50 459
Deletion	28		*krfA*	Frameshift mutation and large deletion	51 359
Deletion	1	G	*KorF*	Frameshift mutation at 99 of 176	52 414
Polymorphism	1	G to A	*klaC*	Gly277Ala	55 232

### Sequencing and analysis of pUZ8002

**Fig. 1.**
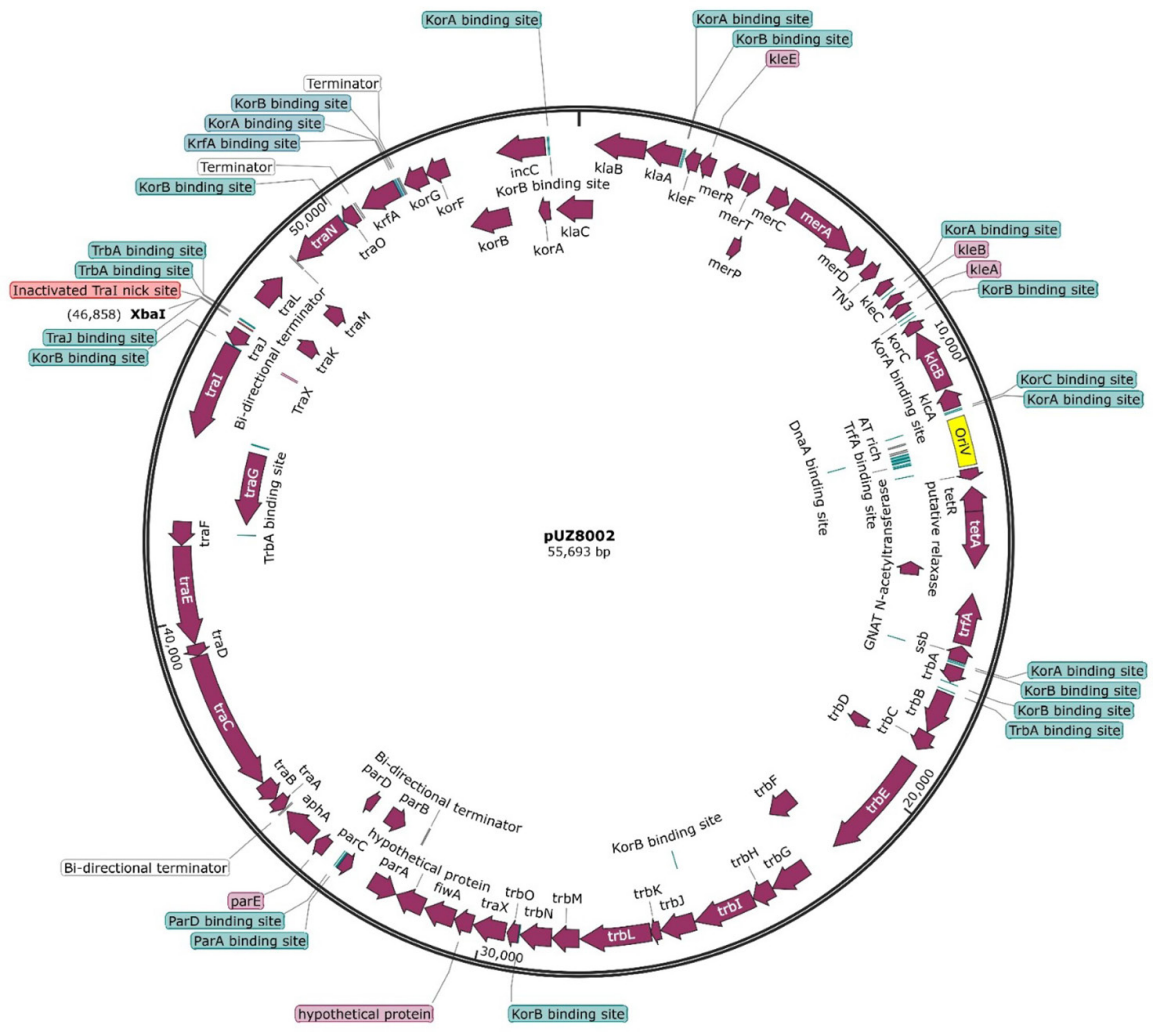
Map of pUZ8002 plasmid. Annotated first by Prokka [25] and manually curated with additional annotations added from RK2 annotation (GenBank BN000925.1) [21,27]. All ORFs were checked manually using blastp.

Plasmid DNA was extracted from *E. coli* ET12567 containing pUZ8002, the standard strain distributed in the *Streptomyces* community for intergeneric conjugation. *E. coli* DH5α (*recA1* and *endA1*) was transformed with this plasmid preparation (with selection on kanamycin – 50 µg ml^−1^) and the resulting pUZ8002-containing colonies were grown overnight and pUZ8002 extracted. The plasmid DNA was sequenced by Plasmidsaurus (Oregon, USA) and the resulting sequence data were assembled as a plasmid map of 55693 bp with 30.6× coverage. Prokka [25] analysis identified 69 ORFs in the sequence and additional annotations, including sites shown to be bound by DNA-binding proteins (KorA, KorB, TrfA, DnaA, IHF, TrbA, ParA, ParD and TraJ) and transcriptional terminators were added to the annotation from the previously published IncP group plasmid RK2 sequence [21,27]. A summary plasmid map is shown in Fig. 1.

Our sequence largely agrees with the previously deposited pUZ8002 sequence (GenBank accession: NZ_MN602278.1). Two differences were detected. Firstly, there is a single base deletion at the extreme 3′ end of the *ssb* gene that encodes a single-stranded DNA-binding protein. This mutation results in no change in amino acid sequence except a conversion of the stop codon (underlined) TAAG to TAG. The second difference is a deletion of a guanine, causing a frameshift mutation at codon 175 of 184 of the *tetR* gene. The sequencing quality score for both sites was above 90%, suggesting it is likely not due to a sequencing error.

### Comparison to RK2

Although pUZ8002 is derived from pUZ8 backbone, there is no available nucleotide sequence for pUZ8, instead here we compared pUZ8002 to the well-studied RK2. Our assumption is that many of the changes will also be evident when comparing RK2 and parental plasmid pUZ8. Comparison of the pUZ8002 sequence to the published RK2 sequence shows 30 differences. These are summarised in Table 1. Particularly noteworthy is the loss of two large transposable elements in pUZ8002 that are present in the RK2 sequence. Firstly, a 4 949 bp Tn1 type transposon carrying the *bla* gene for β-lactam antibiotic resistance, which had inserted in the *klcB* gene (encoding a hypothetical protein of unknown function), and the loss of this Tn1 transposon and beta-lactam resistance has also been demonstrated for the parental plasmid pUZ8 [24]. Secondly, a 2 939 bp IS21 type transposon has inserted into the *aphA* gene [APH(3′)-I aminoglycoside O-phosphotransferase], which confers resistance to kanamycin and other related aminoglycoside antibiotics [31]. The RK2 plasmid is kanamycin resistant [32] despite the IS21 transposon insertion in RK2 between codons 7 and 8. A possible explanation for this is an alternative start codon (GTG) at codon 21 (of 277) in *aphA*, which may result in a truncated but still functional AphA, with transcription potentially driven by an outward-facing promoter in the transposon.

Additionally, in pUZ8002 (and likely in pUZ8) a 4 207 bp Tn3 transposon had inserted itself into the *kleD* gene (predicted to encode a DNA-binding protein). This transposon carries the *mer* locus, an ancient operon that confers resistance to inorganic mercury and other heavy metal stresses [33]. This again supports previous observations that pUZ8 grants resistance to mercury while RK2 does not [24]. The tetracycline resistance genes *tetR* and *tetA* have six mutations in pUZ8002 when compared to the published RK2 sequence. Indeed, our sequence has the additional frameshift mutation in *tetA* that is absent from both RK2 and the pUZ8002 sequence previously deposited in GenBank (accession: NZ_MN602278.1). Without sequencing pUZ8 it is not clear if these mutations are of environmental origin or from the lab, as ET12567 (the donor strain for *Streptomyces* conjugations) is inherently resistant to 50 µg ml^−1^ tetracycline in LB at 37°C, alleviating selective pressure for the *tet* genes.

## Summary

Although pUZ8002 is one of the most widely used plasmids in *Streptomyces* molecular biology, information regarding it was scarce. Here we present the plasmid origins from pUZ8 and the construction methodology, and highlight the key features. Ready access to this information will aid in future development and troubleshooting of *E. coli–Streptomyces* spp. intergenetic conjugations, in addition to preserving an accurate history of *Streptomyces* genetic research.

## Abbreviations

BGC: biosynthetic gene cluster
IncP: incompatability group p
ORF: open reading frame
ori: origin of replication
oriT: origin of transfer
